# Difference of geographic distributions of the Chinese patients with prion diseases in the permanent resident places and referring places

**DOI:** 10.1080/19336896.2022.2080921

**Published:** 2022-05-30

**Authors:** Kang Xiao, Ming-Fan Pang, Yue-Qiao Zhao, Li-Ping Gao, Yue-Zhang Wu, Yuan Wang, Qi Shi, Xiao-Ping Dong

**Affiliations:** a State Key Laboratory for Infectious Disease Prevention and Control, Collaborative Innovation Center for Diagnosis and Treatment of Infectious Diseases (Zhejiang University), National Institute for Viral Disease Control and Prevention, Chinese Center for Disease Control and Prevention, Beijing, Shanghai, China; b Center for Global Public Health, Chinese Center for Disease Control and Prevention, Beijing, Shanghai, China; c China Academy of Chinese Medical Sciences, Beijing, China; d Center for Biosafety Mega-Science, Chinese Academy of Sciences, Wuhan, Hubei, China; e Shanghai Institute of Infectious Disease and Biosafety, Fudan University, Shanghai, China

**Keywords:** Prion disease, Creutzfeldt-Jakob disease, surveillance, case referring, geographic difference

## Abstract

Human prion diseases (PrDs) are a group of transmissible neurodegenerative diseases that can be clarified as sporadic, genetic and iatrogenic forms. In this study, we have analysed the time and geographic distributions of 2011 PrD cases diagnosed by China National Surveillance for Creutzfeldt-Jakob disease (CNS-CJD) since 2006, including 1792 sporadic CJD (sCJD) cases and 219 gPrD cases. Apparently, the cases numbers of both sCJD and gPrD increased along with the surveillance years, showing a stepping up every five years. The geographic distributions of the PrDs cases based on the permanent residences were wide, distributing in 30 out of 31 provincial-level administrative divisions in Chinese mainland. However, the case numbers in the provincial level varied largely. The provinces in the eastern part of China had much more cases than those in the western part. Normalized the case numbers with the total population each province revealed higher incidences in six provinces. Further, the resident and referring places of all PrD cases were analysed, illustrating a clear concentrating pattern of referring in the large metropolises. Five provincial-level administrative divisions reported more PrD cases from other provinces than the local ones. Particularly, BJ reported not only more than one-fourth of all PrDs cases in Chinese mainland but also 3.64-fold more PrDs cases from other provinces than its local ones. We believed that good medical resources, well-trained programmes and knowledge of PrDs in the clinicians and the CDC staffs contributed to well-referring PrD cases in those large cities.

## Introduction

Human prion diseases (PrDs) are a group of fatal and transmissible encephalopathies characterized with spongiform degeneration neuropathologically. PrDs are classified aetiologically as sporadic, inherited and acquired forms [1,2]. Sporadic Creutzfeldt-Jakob disease (sCJD) is the most frequent form accounting for approximately 85% of PrDs. Inherited or genetic PrDs (gPrDs) have different clinical terms, such as genetic CJD (gCJD), fatal familial insomnia (FFI) and Gerstmann-Sträussler-Scheinker syndrome (GSS), showing different clinical and neuropathological features [2–4]. All gPrDs, accounting for 5–15% of all PrDs, are directly linked with the different point mutations or different numbers of the insertions/deletions of octarepeats in the host *PRNP* gene encoding PrP protein. Acquired PrDs consist of iatrogenic CJD (iCJD) and variant CJD (vCJD), which have definite infectious sources from either human or cattle PrDs. The etiologic agent for PrDs is ‘prion’, a pathological protein (PrP^Sc^) showing the identical amino acid (aa.) sequence to a normal cell surface protein (PrP^C^) but different conformational structure [5,6].

Compared with other neurodegenerative diseases, such as Alzheimer disease (AD) and Parkinson disease (PD), the morbidity of PrDs are very low, roughly 1–2 case/million/year worldwide [3,7]. Clinically, PrDs are usually not easy to be recognized and identified, because not only of unspecific clinical manifestations but also of lacking the knowledge of PrDs. Before the outbreak of bovine spongiform encephalopathy (BSE) in 1986 and emerging of vCJD in 1996 in UK and other European countries [8–12], the identification and diagnosis of PrDs were infrequent, and even blank in many countries. Since then, more and more countries have conducted or re-conducted the surveillance work for PrDs, which makes PrDs or CJD be widely recognized. Despite rapid progressions in clinical examination, e.g., MRI, and laboratory tests, e.g., RT-QuIC, the definite diagnosis of PrDs still depends on brain biopsy and autopsy [3].

European countries conducted CJD surveillance relatively early. In the early of 1990ʹs, a network, EUROCJD, covering dozens of European countries, was set up [13]. Later, a broader network had been conducted, which was named NEWROCJD, covering more countries of Europe, North and South America, East Asia. In the early of 2000ʹs, China started to set up the national CJD surveillance and join into the worldwide surveillance system [14]. More than 2000 cases of PrDs (sCJD, gCJD, FFI and GSS) in Chinese mainland have been identified via the China National Surveillance for CJD (CNS-CJD) [15]. In this study, the geographic features of Chinese PrD patients were analysed. A considerable difference in the resident and referring places of Chinese PrDs cases was proposed.

## Materials and methods

### China national surveillance for CJD (CNS-CJD)

CNS-CJD is one of dozens of the national surveillance systems for communicable and non-communicable diseases, as well as other public health events led by Chinese Center for Disease Control and Prevention (China CDC). CNS-CJD started in 2006 formally covering 12 provinces at the beginning, including the local provincial CDCs and 1 to 2 large hospitals [14,16]. Later, all hospitals and CDCs in Chinese mainland can refer the suspected PrD cases directly to CNS-CJD [15]. The surveillance program was approved by the Ethical Review Committee of China CDC. Based on the surveillance documents, the clinical data and specimens of the suspected patients were collected by the clinicians of the local hospitals, while the epidemiological data were collected by the staffs of provincial CDCs. The follow-up survey was performed by either the staffs of China CDC or local CDCs. The collected specimens were transferred to the national reference laboratory for human prion disease in China CDC to conduct the laboratory tests, including Western blot for cerebrospinal fluid (CSF) 14-3-3, *PRNP* gene amplification and sequencing, neuropathological and PrP^Sc^ assays. CSF RT-QuIC were also established in 2017 and was partially applicated in 2019. All laboratory tests were conducted according to the established SOP. The final diagnosis for the suspected patient was made by an expert team consisting of neurologists, epidemiologists and laboratory staff, according to Diagnostic Criteria for CJD (WS/T 562–2017) [17] issued by National Commission of Health, which was similar to the most recent CJD diagnostic criteria [18].

### Data collection

All diagnosed PrD cases from January 2006 to June 2020 from CNS-CJD were included in this study. sCJD cases comprised definite (7 cases), probable (1520 cases) and possible (264 cases) ones. gPrD cases consisted of 18 subtypes of gPrDs with various point-mutations and insertions/deletions in the octarepeat region of *PRNP*, which were gCJD (148 cases), FFI (56 cases) and GSS (15 cases). The resident provinces of the PrD cases were determined by their registered permanent resident places. The referring provinces of the PrD patients were the provincial-level administrative divisions of the hospitals or provincial CDCs referring the suspected PrDs cases to CNS-CJD. The population of every 31 provincial-level administrative divisions (4 municipalities, 5 autonomous regions and 22 provinces) were cited from the official data issued in 2019 [19].

## Results

### Increases of diagnosed sCJD and gPrD cases along with the surveillance years

**Figure 1.**
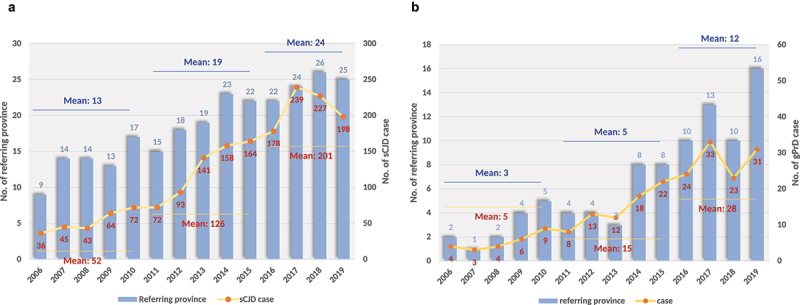
The numbers of the diagnosed PrD cases and the referring provinces from 2006 to 2019. **A**. sCJD cases. **B**. gPrD cases. Yellow curves with Orange points represent the numbers of PrD cases (showing in right Y axis). Light blue columns represent the numbers of referring provinces (showing in left Y axis). The surveillance years are indicated in X axis.

Since 2006, we conducted surveillance work in some provinces of Chinese mainland, 1791 sCJD and 219 different gPrD cases have been identified and diagnosed by June 2020. 89.91% and 90.57% of the diagnosed PrD cases performed brain magnetic resonance imaging (MRI) examination and 14-3-3 test, repsectively. CSF RT-QuIC technique was set up in our laboratory in 2017 and was adopted as an available option in the national surveillance network for CJD since 2019. Recently, skin RT-QuIC was also applied in our surveillance system. About 20% of the diagnosed CJD cases in 2019 and 2020 had RT-QuIC data. Both identified sCJD and gPrD cases increased along with the surveillance year. The mean case numbers of sCJD per year during 2006 to 2010, 2011 to 2015 and 2016 to 2019 were 52, 126 and 201 (Figure 1a, yellow line), while the referring provinces also increased with the mean numbers per year during 2006 to 2010, 2011 to 2015 and 2016 to 2019 of 13, 19 and 24 (blue column), respectively. Similarly, the numbers and referring provinces of gPrDs were increased along with the surveillance years, with the mean numbers per year during 2006 to 2010, 2011 to 2015 and 2016 to 2019 of 5, 15 and 28 in case numbers (Figure 1b, yellow line), and 3, 5 and 12 in referring provinces (blue column), respectively.

**Figure 2.**
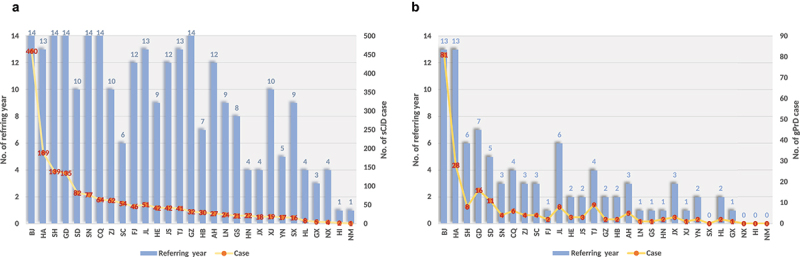
The numbers of the reported PrD cases and the referring years of various provinces. **A**. sCJD cases. **B**. gPrD cases. Yellow curves with Orange points represent the numbers of PrD cases (showing in right Y axis). Light blue columns represent the numbers of referring years (showing in left Y axis). The provinces in abbreviation are indicated in X axis.

In the past 14 surveillance years (2006–2019), six provinces continually referred sCJD cases (Figure 2a, blue column), i.e., Beijing (BJ), Shanghai (SH), Guangdong (GD), Shaanxi (SN), Chongqing (CQ) and Guizhou (GZ). 13 provinces reported sCJD cases in 8 to 13 surveillance years, and 10 provinces reported sCJD cases in 1 to 7 years. Qinghai (QH) and Xizang (XZ, Tibet) did not refer sCJD case. gPrD cases (Figure 2b, blue column) were referred from BJ and Henan (HA) in 13 surveillance years. The other 24 provinces discontinuously reported gPrD cases in 1–7 surveillance years. 5 provinces did not refer gPrD case in the past 14 years, including QH, XZ, Ningxia (NX), Hainan (HI) and Neimenggu (NM). Obviously, BJ reported much more sCJD and gPrD cases than the other provinces during 2006 to 2019 (Figure 2a and 2b, yellow line).

### Considerable difference in the resident and referring places of PrD cases

**Figure 3.**
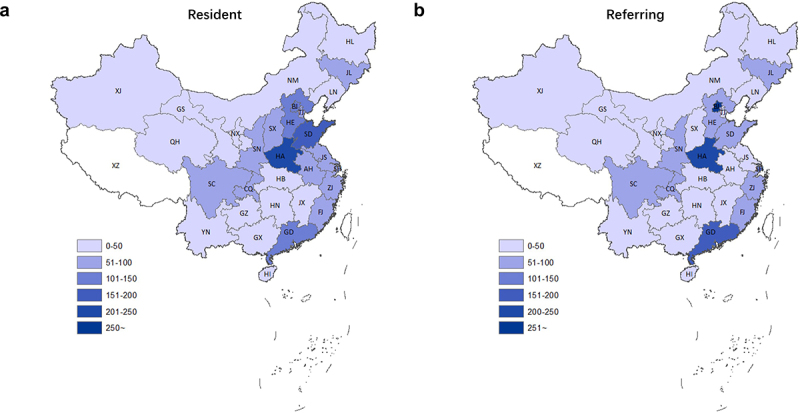
Geographic distributions of PrD cases in the provincial-level administrative divisions of Chinese mainland. **A**. based on the permanent resident places. **B**. based on the referring places.

According to their permanent resident places, the PrD cases distributed widely in 30 out of 31 provinces in Chinese mainland, except Xizang (Table 1). As shown in Figure 3a, majority of PrD cases lived in the eastern part of the provinces in China. Meanwhile, the northern part of the provinces (divided by Yangtze river) had relatively more cases than the southern part. The top 5 most cases provinces were Henan (HA, 245 cases, 12.2%), SD (167 cases, 8.3%), Hebei (HE, 149 cases, 7.4%), GD (131 cases, 6.5%) and BJ (119, 5.6%). Three provinces had referred less than 10 cases, which were NX and NI with 8 cases (0.44%), respectively, as well as QH with only 3 cases (0.15%). 7 PrD cases were from the outside of Chinese mainland, including 6 oversea Chinese and 1 Indian. Further, the ratios of PrD cases based on the population number of the provinces were calculated (Table 1). BJ (5.48), SH (3.60), Tianjin (TJ, 3.33), HA (2.56) and Jilin (JL, 2.21) showed the higher case ratios/mil. of population than 2.0. The case ratios of 17 provinces were between 1.0 and 2.0, while those of 8 provinces were below 1.0 with the lowest in Guangxi (GX) with 0.27.

**Table 1.** Number of cases with prion diseases in the permanent resident places and referring places

Province	Residents (Mil.)	sCJD			gPrD			Total PrD			Ratio of PrD cases/population (Mil.)
		Referring	Resident	Difference	Referring	Resident	Difference	Referring	Resident	Difference	
		Number (%)	Number (%)	Number	Number (%)	Number (%)	Number	Number (%)	Number (%)	Number	
Beijing (BJ)	21.7	468 (26.2%)	106 (5.9%)	362	81 (38.4%)	13 (5.9%)	68	553 (27.5%)	119 (5.9%)	434	5.48
Henan (HA)	95.6	190 (10.6%)	212 (11.8%)	−22	28 (12.8%)	34 (15.1%)	−5	218 (10.8%)	245 (12.2%)	−27	2.56
Shanghai (SH)	24.2	139 (7.8%)	81 (4.5%)	58	8 (3.7%)	6 (2.7%)	2	147 (7.3%)	87 (4.3%)	60	3.60
Guangdong (GD)	111.7	135 (7.5%)	117 (6.5%)	18	16 (7.3%)	14 (6.4%)	2	151 (7.5%)	131 (6.5%)	20	1.17
Shandong (SD)	100.0	86 (4.8%)	143 (8.0%)	−57	12 (5.5%)	24 (11.0%)	−12	98 (4.9%)	167 (8.3%)	−69	1.67
Shaanxi (SN)	38.4	79 (4.4%)	61 (3.4%)	18	5 (2.3%)	5 (2.3%)	0	84 (4.2%)	66 (3.3%)	18	1.95
Chongqing (CQ)	30.5	70 (3.9%)	52 (2.9%)	18	6 (2.7%)	3 (1.4%)	3	76 (3.8%)	55 (2.7%)	21	1.80
Zhejiang (ZJ)	56.6	65 (3.6%)	82 (4.6%)	−17	4 (1.8%)	11 (5.0%)	−7	69 (3.4%)	93 (4.6%)	−24	1.64
Sichuan (SC)	83.0	63 (3.5%)	77 (4.3%)	−14	7 (3.2%)	7 (3.2%)	0	70 (3.5%)	84 (4.2%)	−14	1.01
Fujian (FJ)	39.1	54 (3.0%)	61 (3.4%)	−7	3 (1.4%)	3 (1.4%)	0	57 (2.8%)	64 (3.2%)	−7	1.64
Jilin (JL)	27.2	52 (2.9%)	51 (2.8%)	1	8 (3.7%)	9 (4.1%)	−1	60 (3.0%)	60 (3.0%)	0	2.21
Hebei (HE)	75.2	48 (2.7%)	128 (7.1%)	−80	3 (1.4%)	21 (9.6%)	−18	51 (2.5%)	149 (7.4%)	−98	1.98
Jiangsu (JS)	80.3	45 (2.5%)	69 (3.9%)	−24	2 (0.91%)	5 (2.3%)	−3	47 (2.3%)	74 (3.7%)	−27	0.92
Tianjin (TJ)	15.6	41 (2.3%)	41 (2.3%)	0	9 (4.1%)	11 (5.0%)	−2	50 (2.5%)	52 (2.6%)	−2	3.33
Guizhou (GZ)	35.8	35 (2.0%)	40 (2.2%)	−5	2 (0.91%)	4 (1.8%)	−2	37 (1.8%)	44 (2.2%)	−7	1.23
Hubei (HB)	59.0	30 (1.7%)	36 (2.0%)	−6	2 (0.91%)	4 (1.8%)	−2	32 (1.6%)	40 (2.0%)	−8	0.68
Anhui (AH)	62.5	27 (1.5%)	64 (3.6%)	−37	5 (2.3%)	9 (4.1%)	−4	32 (1.6%)	73 (3.6%)	−41	1.17
Liaoning (LN)	43.7	25 (1.4%)	44 (2.5%)	−19	1 (0.46%)	3 (1.4%)	−2	26 (1.3%)	47 (2.3%)	−21	1.08
Gansu (GS)	26.3	23 (1.3%)	39 (2.2%)	−16	2 (0.91%)	4 (1.8%)	−2	25 (1.2%)	43 (2.1%)	−18	1.63
Hunan (HN)	68.6	22 (1.2%)	26 (1.5%)	−4	2 (0.91%)	1 (0.46%)	1	24 (1.2%)	27 (1.3%)	−3	0.39
Jiangxi (JX)	46.2	20 (1.1%)	36 (2.0%)	−16	3 (1.4%)	9 (4.1%)	−6	23 (1.1%)	45 (2.2%)	−22	0.97
Xinjiang (XJ)	24.4	19 (1.1%)	24 (1.3%)	−5	1 (0.46%)	2 (0.91%)	−1	20 (0.99%)	26 (1.3%)	−6	1.07
Yunnan (YN)	48.0	17 (0.95%)	24 (13.4%)	−7	2 (0.91%)	2 (0.91%)	0	19 (0.94%)	26 (1.3%)	−7	0.54
Shanxi (SX)	37.0	16 (0.89%)	58 (3.2%)	−42	0 (0.0%)	3 (1.4%)	−3	16 (0.80%)	61 (3.0%)	−45	1.65
Heilongjiang (HL)	37.9	8 (0.44%)	45 (2.5%)	−37	2 (0.91%)	4 (1.8%)	−2	10 (0.50%)	49 (2.4%)	−39	1.29
Guangxi (GX)	48.9	5 (0.28%)	12 (0.67%)	−7	1 (0.46%)	1 (0.46%)	0	6 (0.30%)	13 (0.65%)	−7	0.27
Ningxia (NX)	6.8	4 (0.22%)	7 (0.39%)	−3	1 (0.46%)	1 (0.46%)	0	5 (0.25%)	8 (0.40%)	−3	1.18
Hainan (HI)	9.3	3 (0.17%)	6 (0.33%)	−3	1 (0.46%)	2 (0.91%)	−1	4 (0.20%)	8 (0.40%)	−4	0.86
Neimenggu (NM)	25.3	2 (0.11%)	33 (1.8%)	−31	0 (0.0%)	6 (2.7%)	−6	2 (0.10%)	39 (1.9%)	−37	1.54
Qinghai (QH)	6.0	0 (0%)	2 (0.11%)	−2	0(0.0%)	1 (0.46%)	−1	0 (0.0%)	3 (0.15%)	−3	0.50
Outside	/	/	6 (0.33%)	/	/	1 (0.46%)	/	/	7 (3.5%)	/	/
Total	/	1792	1792	/	219	219	/	2011	2011	/	/

Analysis of the referring case numbers showed different scenarios (Table 1, Figure 3b). 553 PrD cases were reported from BJ that accounted for 27.5% of all PrD cases. The other 4 provinces with higher referring case numbers were HA (218, 10.8%), GD (151, 7.5%), SH (147, 7.3%) and SD (98, 4.9%). Calculating the differences between the referring case numbers and resident case numbers showed five provinces having more referring cases than its resident ones, including BJ with 424 cases more, SH with 60 cases more, CQ with 21 cases more, GD with 20 cases more and SN with 18 cases more. JL had equal referring and resident case numbers. The rest provinces referred less cases than their resident ones.

### BJ, SH and other metropolises refer more PrD cases from other provinces

**Figure 4.**
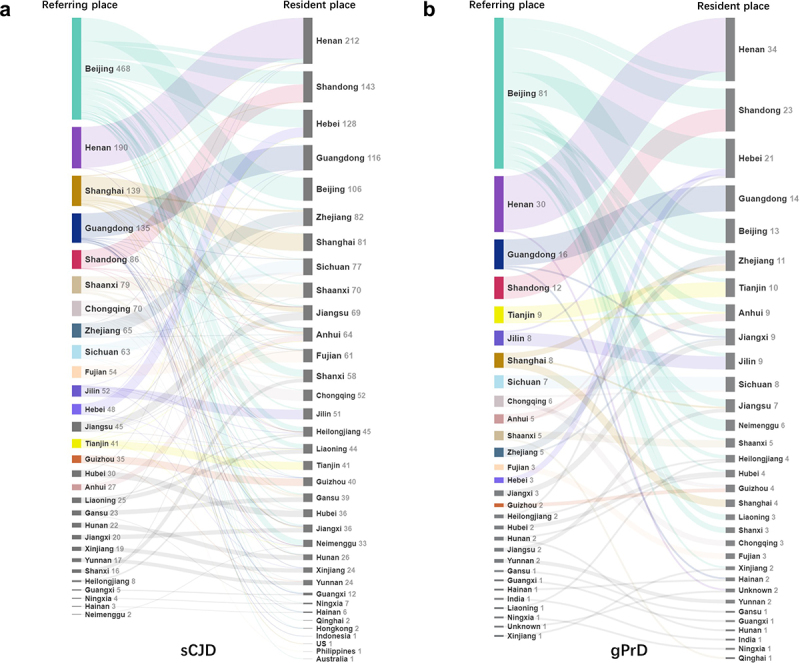
Distributions of PrD cases based on the resident places (left) and referring places (right) of various provinces in Chinese mainland. **A**. sCJD cases. **B**. gPrD cases.

We further analysed the resources of the PrD cases based on their permanent residences in their referred and diagnosed places. As shown in Figure 4a, BJ received and referred the vast majority of sCJD patients (362 cases) from other provinces. Those sCJD patients came from 28 provincial-level administrative divisions in Chinese mainland, as well as two oversea Chinese sCJD cases. Most sCJD case were from northern part of China, such as HE (81cases), SD (60 cases), Shanxi (SX, 34 cases), NM (27 cases), Heilongjiang (HL, 22 cases), Liaoning (LN, 21 cases), HA (20 cases), etc. SH referred 58 sCJD cases from other provinces and 1 oversea case. Most cases came from neighbour provinces, e.g., Jiangsu (JS, 23 cases), Anhui (AH, 11 cases), Zhejiang (ZJ, 8 cases), Jiangxi (JX, 5 cases), etc. GD referred 18 sCJD cases from neighbour provinces, such as GX (5 cases), JX (4 cases), HI (2 cases), Fujian (FJ, 2 cases). GD also reported 2 oversea Chinese cases. CQ also reported 18 sCJD cases from the neighbour provinces, such as Sichuan (SC, 11 cases), Yunnan (YN, 3 cases), Hubei (HB, 2 cases), GZ (2 cases). SN referred 18 cases from other provinces, mostly from neighbour province Gansu (GS, 10 cases) and SX (7 cases).

The referring pattern of gPrDs based on their resident places were similar as that of sCJD. In addition to 13 local gPrD cases, BJ referred 68 cases from other provinces, e.g., HE (16 cases), SD (11 cases), NM (6 cases), HA (5 cases), AH (4 cases), JS (4 cases), JX (4 cases), etc. (Figure 4b). Other 4 provinces reported a few gPrD cases from other provinces besides of local ones, including CQ (3 cases), SH (2 cases), GD (2 cases) and Hunan (HN, 1 case). Obviously, the hospitals in BJ, SH and other metropolises identified and referred more PrD cases from other provinces.

## Discussion

As a rare disease, less than 10 Chinese PrD or CJD cases had been described in the literatures in 1990ʹs. More and more PrD cases have been identified and diagnosed since 2006 we conducted CNS-CJD, particularly in the past recent years [15,20]. Small numbers of definite sCJD cases and overnumbered probable sCJD cases in CNS-CJD are closely related with the extremely low rate of autopsy in China. Moreover, more and more provinces actively refer PrD cases to CNS-CJD along with the surveillance years. Our data here propose that about half of the provinces in Chinese mainland continually reported PrD cases in the past 10 years. It reflects that the clinical recognition capacity for PrD in Chinese mainland has been improved markedly after the implementation of CNS-CJD.

We have noticed the obvious diversity in geographic distribution of the PrD cases based on their permanent addresses among the provinces. The case numbers in the provinces locating at the western part of China are much less. After normalized with the total population of each province, the geographic patterns of PrD cases has changed in a certain degree. More referred cases are concentrated in the large metropolises, such as BJ, SH TJ and CQ, with higher ratio of urban population, better medical resources and economic situation, which is most likely reflecting the wide and good knowledge of PrD among the physicians in those large cities.

Our data also figure out that five provincial-level administrative divisions, including BJ, SH, GD, CQ and SN, have referred more cases of PrDs coming from other provinces than those from locals. JL province has referred the same numbers of PrD cases as the local ones. The referred case numbers of the rest provinces in Chinese mainland are less than the local ones. This scenario demonstrates excessive contributions of those five provinces to identifying and referring PrD cases. As the capital of China, BJ city has unparalleled medical resources. Ordinarily, about half of the patients in the large hospitals in BJ come from other provinces [21], particularly the patients of rare and difficult-diagnosed diseases. Previously, we had analysed the CJD surveillance activity in BJ from 2006 to 2013, proposing that the ratio of the diagnosed PrD cases from local and other provinces is 1:3.07 [21]. Such ratio maintains unchanged, even slightly increases, by June 2020 from the data in this study (1:3.65), strongly indicating the continually great role of BJ in the recognition for PrDs in CNS-CJD. On the other point, the PrD cases reported from BJ show a wide geographic distribution covering 28 different provinces, despite of large amount of cases from the neighbour provinces. It reflects that the BJ’s medical system supplies the medical services not only for BJ’s people but also for the people outside of BJ.

In addition to BJ, the other four provincial-level administrative divisions (SH, GD, CQ and SH) also contribute more PrD cases from other provinces. Unlike BJ, the outside PrD cases reported from those four provinces mainly locate at the neighbour provinces. Like BJ, SH and CQ are also metropolises with high level of medical resources, making them capable of receiving hundreds of patients from the neighbour provinces. Guangzhou and Xi’an cities are the capital cities of provinces GD and SN, respectively. There are more than two medical universities and dozens of large hospitals in those two large cities. Apparently, good medical resources in the large cities are one of the major elements for identifying and referring PrDs.

During the implementation of CNS-CJD, different trainings from clinical to laboratory have been conducted by China CDC almost every surveillance year. The trainees come from both the CDC sections and the department of neurology in hospitals. In some places, e.g., BJ and SH, the provincial-level CDCs have also conducted further PrD trainings to more physicians from local hospitals. We believe that those training programs have enhanced the capacity of the clinicians for PrDs. Actually, the numbers of hospitals referring PrD cases in BJ and SH are much more than the other cities (data not shown). As a huge and developing countries, the medical resources in Chinese mainland is markedly unbalance. Both numbers of referred PrD cases and referring hospitals in the western part of China are less. Further practicable training programs for the staffs in those provinces are critical.

Our data here has illustrated the similar profiles in referring sCJD and gPrD cases among the provinces that BJ city has referred more than one-third of all diagnosed gPrD cases in Chinese mainland. Besides of FFI [13,22], other types of gPrDs (gCJD and GSS) usually display undistinguishable clinical manifestations as sCJD, except that the onset-ages of gPrDs are usually young [23,24]. In fact, the vast majority of gCJD and GSS were referred as sCJD before *PRNP* sequencing. Notably, HA province referred obviously more FFI cases than other provinces, since more than one-third of Chinese FFI cases lived in HA [22]. Good knowledge of FFI possibly makes the local neurologists more sensitive for the patients with unexplainable sleeping problem.

## Conclusions

According to our study, the cases numbers of both sCJD and gPrD increased along with the surveillance years, with a wide geographic distribution in Chinese mainland. However, the case numbers in the eastern provinces were much more than those in the western provinces. Our study indicated that good medical resources, well training programs and knowledge of PrDs in the clinicians and the CDC staffs contributed to well-referring PrD cases in those large cities.
